# Fuel for the Work Required: A Theoretical Framework for Carbohydrate Periodization and the Glycogen Threshold Hypothesis

**DOI:** 10.1007/s40279-018-0867-7

**Published:** 2018-02-16

**Authors:** Samuel G. Impey, Mark A. Hearris, Kelly M. Hammond, Jonathan D. Bartlett, Julien Louis, Graeme L. Close, James P. Morton

**Affiliations:** 1 Research Institute for Sport and Exercise Sciences, Liverpool John Moores University, Tom Reilly Building, Byrom St Campus, Liverpool, L3 3AF UK; 2 Institute of Sport, Exercise and Active Living (ISEAL), Victoria University, Footscray Park, Ballarat Road, Melbourne, VIC 8001 Australia

## Abstract

Deliberately training with reduced carbohydrate (CHO) availability to enhance endurance-training-induced metabolic adaptations of skeletal muscle (i.e. the ‘train low, compete high’ paradigm) is a hot topic within sport nutrition. Train-low studies involve periodically training (e.g., 30–50% of training sessions) with reduced CHO availability, where train-low models include twice per day training, fasted training, post-exercise CHO restriction and ‘sleep low, train low’. When compared with high CHO availability, data suggest that augmented cell signalling (73% of 11 studies), gene expression (75% of 12 studies) and training-induced increases in oxidative enzyme activity/protein content (78% of 9 studies) associated with ‘train low’ are especially apparent when training sessions are commenced within a specific range of muscle glycogen concentrations. Nonetheless, such muscle adaptations do not always translate to improved exercise performance (e.g. 37 and 63% of 11 studies show improvements or no change, respectively). Herein, we present our rationale for the glycogen threshold hypothesis, a window of muscle glycogen concentrations that simultaneously permits completion of required training workloads and activation of the molecular machinery regulating training adaptations. We also present the ‘fuel for the work required’ paradigm (representative of an amalgamation of train-low models) whereby CHO availability is adjusted in accordance with the demands of the upcoming training session(s). In order to strategically implement train-low sessions, our challenge now is to quantify the glycogen cost of habitual training sessions (so as to inform the attainment of any potential threshold) and ensure absolute training intensity is not compromised, while also creating a metabolic milieu conducive to facilitating the endurance phenotype.

## Key Points

**Table:** 

Periodically completing endurance training sessions (e.g. 30–50% of training sessions) with reduced carbohydrate (CHO) availability modulates the activation of acute cell signalling pathways (73% of 11 studies), promotes training-induced oxidative adaptations of skeletal muscle (78% of 9 studies) and, in some instances, improves exercise performance (although only 37% of 11 studies demonstrated performance improvements).
We propose the presence of a muscle glycogen threshold whereby exceeding a critical absolute level of glycogen depletion during training is especially potent in modulating the activation of acute and chronic skeletal muscle adaptations associated with ‘train low’.
Future research should attempt to quantify the glycogen and CHO cost of endurance athletes’ typical training sessions so as to increase our understanding of the exercise conditions that may elicit the proposed glycogen threshold and thereby inform practical application of ‘fuel for the work required’ paradigm.

## Introduction

The principle of ensuring sufficient carbohydrate (CHO) availability before, during and after training and competition is widely recognized as the fundamental nutritional priority for athletic populations. Indeed, the foundation of current sport nutrition guidelines [1] were developed by Scandinavian researchers in the late 1960s with the introduction of the muscle biopsy technique [2–5] and the classical ‘super-compensation’ model of CHO loading. In another landmark study in 1981, Sherman and colleagues [6] observed similar magnitudes of glycogen super-compensation with a less severe protocol (i.e., without the exhaustive exercise and CHO restriction phase), whereby several days of a combined exercise taper and moderate CHO intake (e.g. 5 g kg^−1^ body mass) is followed by 3 days of higher CHO intake (8 g kg^−1^ body mass). While these data have practical application from a precompetition perspective, one of the most overlooked components of this study is that no differences in half-marathon running performance (as completed on an outdoor 220 m running track) were observed between trials, despite differing pre-exercise muscle glycogen status. The authors allude to this finding when discussing their data:

“The performance times indicate that carbohydrate loading was of no benefit to performance during the 20.9 km run. In fact, performance times were actually slower in the trials with higher levels of muscle glycogen. This suggests that anything above a minimal level of muscle glycogen is unnecessary for performance of a given intensity and duration. More importantly, the practical question may not be how much can I super-compensate but rather, does my diet contain enough carbohydrate to maintain adequate stores of muscle glycogen on a day-to-day basis for training and performance runs?” (p. 117).

**Fig. 1:**
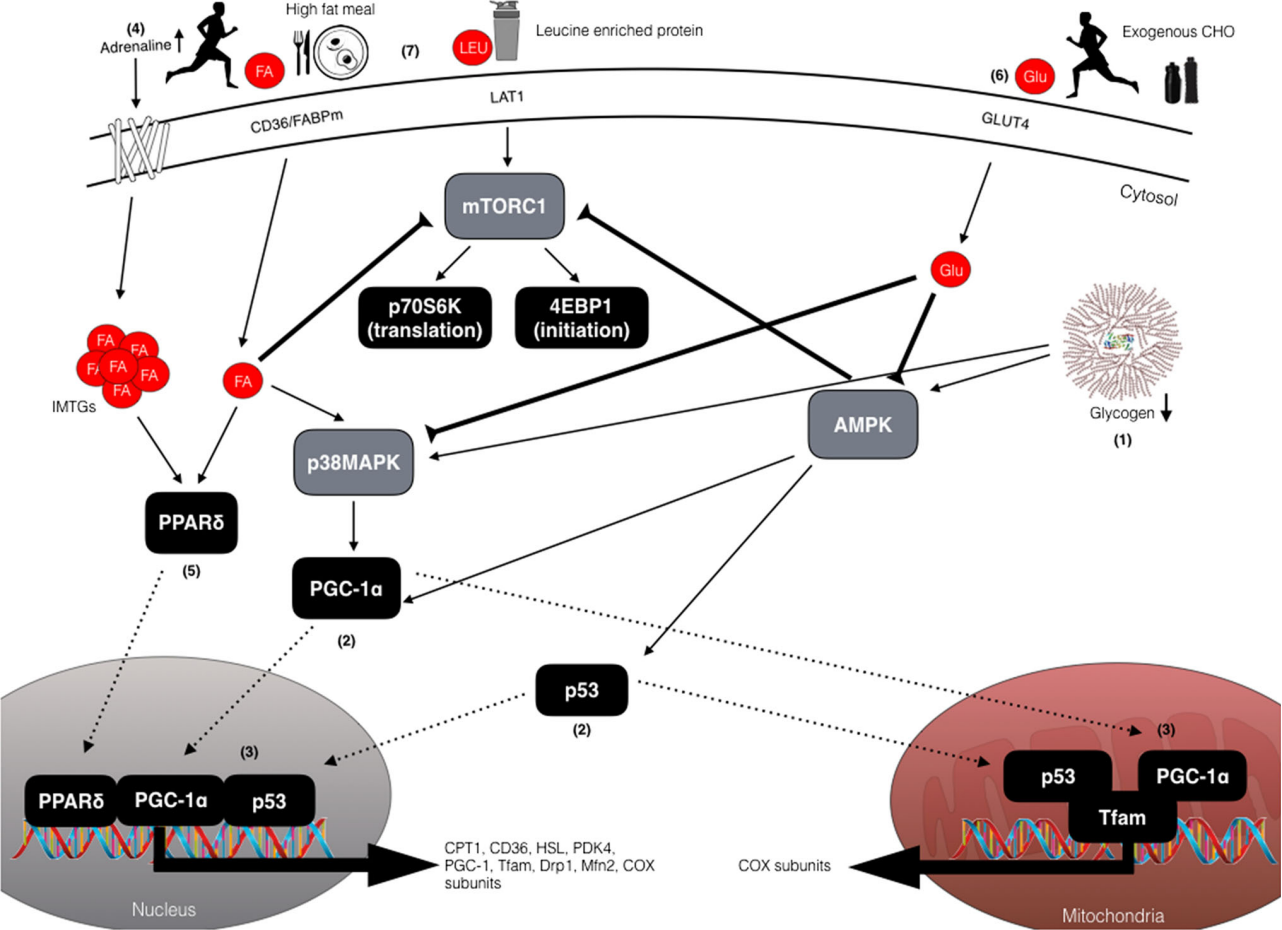
Schematic overview of the potential exercise-nutrient-sensitive cell signalling pathways regulating the enhanced mitochondrial adaptations associated with training with low CHO availability. (1) Reduced muscle glycogen enhances both AMPK and p38MAPK phosphorylation that results in (2) activation and translocation of PGC-1α and p53 to the mitochondria and nucleus. (3) Upon entry into the nucleus, PGC-1α co-activates additional transcription factors (i.e. NRF1/2) to increase the expression of COX subunits and Tfam, as well as autoregulating its own expression. In the mitochondria, PGC-1α co-activates Tfam to coordinate regulation of mtDNA, and induces expression of key mitochondrial proteins of the electron transport chain, e.g. COX subunits. Similar to PGC-1α, p53 also translocates to the mitochondria to modulate Tfam activity and mtDNA expression, and to the nucleus where it functions to increase expression of proteins involved in mitochondrial fission and fusion (DRP-1 and MFN-2) and electron transport chain proteins. (4) Exercising in conditions of reduced CHO availability increases adipose tissue and intramuscular lipolysis via increased circulating adrenaline concentrations. (5) The resulting elevation in FFA activates the nuclear transcription factor, PPARδ, to increase expression of proteins involved in lipid metabolism, such as CPT1, PDK4, CD36 and HSL. (6) However, consuming pre-exercise meals rich in CHO and/or CHO during exercise can downregulate lipolysis (thereby negating FFA-mediated signalling), as well as reducing both AMPK and p38MAPK activity, thus having negative implications for downstream regulators. (7) High-fat feeding can also modulate PPARδ signalling and upregulate genes with regulatory roles in lipid metabolism (and downregulate CHO metabolism), although high-fat diets may also reduce muscle protein synthesis via impaired mTOR-p70S6K signalling, despite feeding leucine-rich protein. *4EBP1* eukaryotic translation initiation factor 4E-binding protein 1, *AMPK* AMP-activated protein kinase, *CHO* carbohydrate, *CD36* cluster of differentiation 36, *COX* cytochrome c oxidase, *CPT1* carnitine palmitoyltransferase 1, *Drp1* dynamin-related protein 1, *FA* fatty acid, *FABP* fatty acid binding protein, *GLU* glucose, *GLUT4* glucose transporter type 4, *HSL* hormone-sensitive lipase, *IMTG* intramuscular triglycerides, *LAT1* large neutral amino acid transporter, *LEU* leucine, *Mfn2* mitofusion-2, *mTORC1* mammalian target of rapamycin complex 1, *p38MAPK* p38 mitogen-activated protein kinase, *p53* tumor protein 53,  *p70S6K* ribosomal protein S6 kinase, *PDK4* pyruvate dehydrogenase kinase 4, *PGC*-*1α* peroxisome proliferator-activated receptor gamma coactivator 1-α, *PPARδ* peroxisome proliferator-activated receptor, *Tfam* mitochondrial transcription factor A

In addition to competitive performance, the same lead author subsequently challenged the concept that insufficient daily CHO intake on consecutive training days reduces muscle glycogen availability to a level that impairs training capacity [7, 8]. While such sentiments are undoubtedly dependent on the prescribed training workloads, almost 30 years later the above interpretation and insight appears more relevant than ever. Indeed, with the introduction of molecular biology techniques into the sport and exercise sciences, we are now in the era of ‘nutrient-gene interactions’ whereby the nutritional modulation of endurance training adaptation is a contemporary area of investigation. Since the seminal work of Hansen et al. [9] suggested that some adaptations to physical activity may require a ‘cycling’ of muscle glycogen stores, the role of glycogen availability as a training regulator has gained increased popularity among athletic circles [10]. This body of work has largely focused on the efficacy of the periodic ‘train low (smart), compete high’ paradigm whereby selected training sessions are deliberately completed with reduced CHO availability so as to activate molecular pathways that regulate skeletal muscle adaptation (see Fig. 1). In contrast, it is recommended that both key training sessions and competition always be undertaken with high CHO availability so as to promote performance and recovery [11, 12]. Nonetheless, the optimal approach for which to practically apply periods of ‘train low’ (now commonly referred to as CHO periodization) into an overall athletic training programme is not currently understood.

Accordingly, the aim of this article is to present a contemporary overview of CHO periodization strategies for training from both a theoretical and practical perspective. We begin by outlining the effects of various train-low paradigms on modulating cell signalling pathways, training adaptations and exercise performance. We then present our rationale for the ‘glycogen threshold hypothesis’, a window of absolute muscle glycogen concentration of which training sessions could be commenced within so as to provide a metabolic milieu that is conducive to modulating cell signalling. We close by presenting a practical model of CHO periodization according to the principle of ‘fuel for the work required’. As opposed to chronic periods of CHO restriction, this model suggests that CHO availability should be manipulated day-to-day and meal-by-meal according to the intensity, duration and specific training goals. With this in mind, we define CHO availability as the sum of the endogenous (i.e., muscle and liver glycogen) and exogenous CHO (i.e. CHO consumed before and/or during exercise) that is available to sustain the required training intensity and duration. According to this definition, it is possible to have insufficient CHO availability (even if exercise is commenced with high pre-exercise muscle glycogen stores) if an inadequate dose of CHO is consumed during exercise to sustain the desired workload [13]. Alternatively, it is possible to commence exercise with reduced muscle glycogen yet still be considered to have sufficient CHO availability if the exogenous CHO consumed during exercise permits the completion of the required training intensity and duration [14].

## Carbohydrate (CHO) Restriction Enhances Cell Signalling and Gene Expression, and Modulates Components of Training Adaptation and Exercise Performance

Researchers have used a variety of acute and chronic train-low interventions to investigate the efficacy of CHO restriction and periodization on training adaptations and exercise performance. An overview of specific train-low models is discussed below and relevant experimental details from seminal studies are summarized in Table 1. Additionally, key outcomes from such studies are also categorized under the measures of cell signalling, gene expression, enzymatic changes and performance outcomes, according to positive changes, no change or negative changes (see Table 2). Despite the evidence supporting enhanced skeletal muscle adaptations with CHO restriction, it is noteworthy that such adaptations do not always translate to improved exercise performance.

**Table 1:** Overview of the methodological details and study outcomes of acute and chronic train-low studies according to the relevant train-low paradigm

Reference	Subjects	Duration	Exercise protocol and glycogen status (mmol/kg dw)	Skeletal muscle adaptations	Exercise performance outcomes
Twice per day model					
Hansen et al. [9]	7 Untrained men	10 weeks 5 days × week	Knee extensor exercise. One leg trained 50% of sessions with low glycogen (LOW), while the other trained all sessions with high glycogen (HIGH). Second session glycogen in LOW—pre: 200, post: 100 mmol/kg dw, respectively	Greater increase in CS activity in the LOW condition Increased β-HAD activity in the LOW condition only	Improved TTE for knee extensor exercise
Yeo et al. [17]	14 trained male cyclists/triathletes	3 weeks 4 × week	100 min steady-state cycling (63% PPO) followed by 8 × 5-min intervals at maximal pace either 2 h (LOW) or 24 h (HIGH) later. Pre-interval exercise glycogen—LOW: 256, HIGH: 390. Post-exercise glycogen—LOW: 124, HIGH: 229	Increased CS and β-HAD activity in the LOW condition only Increased COXIV protein content in the LOW condition only	Similar improvements (10%) in 60-min TT for both groups
Morton et al. [18]	23 active men	6 weeks 4 × week	6 × 3-min running (90% VO_2max_). NORM trained once per day, while LOW + PLA and LOW + GLU trained twice per day (every other day). LOW + GLU ingested CHO before and during every second training session. Pre exercise glycogen—LOW: 232 and 253, HIGH: 412 and 387 in the gastrocnemius and vastus lateralis, respectively. Post-exercise glycogen—LOW: 107 and 176, HIGH: 240 and 262 in the gastrocnemius and vastus lateralis, respectively	Greater increase in SDH activity in LOW + PLA compared with LOW + GLU and NORM	Similar improvements in VO_2max_ and YoYoIR2 for all groups
Yeo et al. [23]	12 trained male cyclists/triathletes	Acute exercise	100-min steady-state cycling (63% PPO) followed by 8 × 5-min intervals at maximal pace either 2 h (LOW) or 24 h (HIGH) later. Pre-interval exercise glycogen—LOW: 256, HIGH: 390. Post-exercise glycogen—LOW: 124, HIGH: 229	Greater phosphorylation of AMPK^Thr172^ in LOW	NA
Hulston et al. [19]	14 trained male cyclists	3 weeks 6 × week	90-min cycling at 70% VO_2max_ followed by (2 h apart) HIT (8 × 5 min) in the LOW group. The HIGH group performed alternate days of either steady state or HIT cycling. Acute glycogen status not measured	β-HAD protein content increased in LOW only Increased fat utilization from muscle triglycerides in LOW only	Similar improvements in 60-min TT for both groups
Cochran et al. [22]	10 Active men	Acute exercise	HIT cycling (5 × 4-min at 90–95% heart rate reserve) twice per day (separated by 3 h). One group consumed CHO (2.3 g.kg) between sessions (HIGH), whereas the other group restricted CHO intake (LOW). Pre-pm exercise glycogen—LOW: 256, HIGH: 390. Post-exercise glycogen—LOW: 124, HIGH: 229	Greater phosphorylation of p38MAPK in LOW following pm exercise Similar increase in PGC-1α and COXIV gene expression	NA
Cochran et al. [20]	18 Active men	2 weeks 3 days × week	HIT cycling (5 × 4 min at 60% PPO) twice per day (separated by 3 h). One group consumed CHO (2.3 g kg) between sessions (HIGH), whereas the other group restricted CHO intake (LOW). Acute glycogen status not measured	Similar increase in maximal CS activity and protein content of both CS and COXIV	Greater improvement in 250-kJ TT performance in the LOW group
Fasted training model					
Akerstrom et al. [27]	9 Active men	Acute exercise	2 h one-legged knee extensor exercise (60% *W*_max_) in either a fasted (FAST) or fed (exogenous CHO during; FED) state. Pre-exercise glycogen: 500 mmol/kg dw in both groups. Post-exercise glycogen: 300 and 200 in the FED and FAST states, respectively	Reduced AMPKα2 activity in FED	NA
Lee-Young et al. [49]	9 Active men	Acute exercise	120-min cycling (65% VO_2peak_) exercise in either a fasted (FAST) or fed (exogenous CHO during; FED) state. Pre-exercise glycogen: 500 mmol/kg dw in both groups. Post-exercise glycogen: 150 and 100 in the FED and FAST states, respectively	Similar increases in AMPKα2 activity and AMPKα2^Thr172^ and ACC-β^Ser222^ phosphorylation	NA
De Bock et al. [31]	20 Active men	6 weeks 3 × week	1–2 h cycling (75% VO_2peak_). One group trained in the fasted state (FAST), with the other consuming CHO before and during exercise (FED). Acute glycogen status not measured	FABP increased in the FAST condition only	NA
Nybo et al. [32]	15 untrained men	8 weeks 3–4 × week	3–6 min of high-intensity intervals (70–85% VO_2max_). Subjects received either CHO or PLA during exercise. Acute glycogen status not measured	Greater increases in β-HAD activity and basal muscle glycogen content in the PLA group only	Similar improvements in peak power, VO_2max_ and 15-min TT performance
Van Proeyen et al. [30]	20 active men	6 weeks 4 × week	1–1.5 h cycling (70% VO_2max_). One group trained in the fasted state (FAST), with the other consuming CHO before and during exercise (FED). Acute glycogen status not measured	CS and β-HAD maximal activity increased in the FAST condition only	Similar improvements in 1-h TT performance in both groups
Sleep-low model					
Pilegaard et al. [15]	Study A: 6 active men Study B: 6 active men	Acute exercise Acute exercise	Study A: 1-legged glycogen-depleting exercise followed by 2-legged cycling (2 h at 45% VO_2max_) on the subsequent day. Pre-exercise glycogen—LOW: 337, HIGH: 609. Post-exercise glycogen—LOW: 306, HIGH: 423 Study B: 3 h of 2-legged knee extensor exercise with either NORM or LOW glycogen. Pre-exercise glycogen—LOW: 240, HIGH: 398. Post-exercise glycogen—LOW: 101, HIGH: 153	Study A: Enhanced gene expression of PDK4, LPL and HKII at rest in LOW only Studies A and B: Enhanced gene expression of PDK4 and UCP3 post-exercise in LOW only	NA NA
Wojtaszewski et al. [36]	8 Trained men	Acute exercise	60-min cycling at 70% VO_2peak_ with either LOW or HIGH muscle glycogen (from exercise/diet manipulation the previous day). Pre-exercise glycogen—LOW: 163, HIGH: 909. Post-exercise glycogen—LOW: 150, HIGH: 400	Increased AMPKα2 activity in LOW only Greater phosphorylation of ACC^Ser221^ in LOW	NA
Chan et al. [37]	8 active men	Acute exercise	60-min cycling (70% VO_2peak_) with either HIGH or LOW glycogen (achieved by exercise/diet manipulation the previous evening). Pre-exercise glycogen—LOW: 163, HIGH: 375. Post-exercise glycogen—LOW: 17, HIGH: 102	Greater phosphorylation of p38MAPK in LOW Enhanced gene expression of IL-6 in LOW	NA
Steinberg et al. [21]	7 active men	Acute exercise	60-min cycling at 70% VO_2max_ with either LOW or NORM muscle glycogen. Pre-exercise glycoge—LOW: 150, HIGH: 390. Post-exercise glycogen—LOW: 17, HIGH: 111	Greater AMPKα2 activity, phosphorylation of ACC^Ser221^ and nuclear translocation of AMPKα2 in LOW only Enhanced gene expression of GLUT4 in LOW	NA
Bartlett et al. [38]	8 active men	Acute exercise	HIT running (6 × 3 min at 90% VO_2max_). The LOW group performed glycogen-depleting cycling the night before and restricted CHO overnight. The HIGH group consumed a high-CHO breakfast and CHO during exercise. Pre-exercise glycogen – LOW: 100, HIGH: 500. Post-exercise glycogen—LOW: 80, HIGH: 300	Phosphorylation of ACC^Ser79^ and p53^Ser15^ in LOW only Enhanced gene expression of PGC-1α, PDK4, Tfam and COXIV in LOW	NA
Psilander et al. [24]	10 trained male cyclists	Acute exercise	6 × 10-min cycling (64% VO2_max_) with either HIGH or LOW glycogen (achieved by exercise/diet manipulation 14 h previously). Pre-exercise glycogen—LOW: 166, HIGH: 478. Post-exercise glycogen—LOW: 130, HIGH: 477	Enhanced gene expression of PGC-1α in LOW Increased gene expression of PDK4 and COXIV in LOW only	NA
Lane et al. [39]	7 trained male cyclists	Acute exercise	Evening bout of high-intensity cycling (8 × 5 min at 82.5% PPO) followed by 120-min steady-state cycling (50% PPO) the subsequent morning. The LOW group restricted CHO overnight, whereas the HIGH group consumed a high-CHO diet (4 g.kg BM). Pre-exercise glycogen—LOW: 349, HIGH: 459. Post-exercise glycogen—LOW: 266, HIGH: 338	Greater phosphorylation of ACC^Ser79^ post-AM exercise in LOW Enhanced gene expression of CD36, FABP3 and PDK4 post-AM exercise in LOW	NA
Marquet et al. [40]	21 male triathletes	3 weeks 6 × week	HIT (8 × 5 min cycling at 85% MAP or 6 × 5-min running at individual 10-km intensity) in the evening followed by LIT (60-min cycling at 65% MAP) the subsequent morning. One group consumed CHO between training sessions (HIGH), whereas the other group restricted CHO intake (LOW). Acute glycogen status not measured	NA	Improved 10-km running TT performance and improved TTE cycling (150% peak aerobic power) in the LOW group only
Marquet et al. [41]	11 trained male cyclists	1 week 6 × week	HIT (8 × 5-min cycling at 85% MAP) in the evening followed by LIT (60-min cycling at 65% MAP) the subsequent morning. One group consumed CHO between training sessions (HIGH), whereas the other group restricted CHO intake (LOW). Acute glycogen status not measured	NA	Improved 20-km cycling TT performance in the LOW group only
Recover-low model					
Pilegaard et al. [16]	9 active men	Acute exercise	75-min cycling (75% VO_2max_) followed by 24 h recovery with either HIGH or LOW CHO diet. Glycogen was restored to 576 and 348 with HIGH and LOW CHO diets, respectively, at 24 h	Gene expression of PDK4, UCP3, LPL and CPT1 remained elevated for 8–24 h with CHO restriction post-exercise	NA
Jensen et al. [54]	15 male triathletes	Acute exercise	4-h cycling (56% VO_2max_) followed by 4 h recovery feeding with either HIGH (1 g.kg.h) or LOW (water only) CHO. Post-exercise glycogen—LOW: 234, HIGH: 245. 4-h glycogen—LOW: 264, HIGH: 444	Similar gene expression of PGC-1α, Tfam, NRF-1, COXIV, PDK4, LPL, PPAR, UCP3 and GLUT4 in both groups	NA
High-fat feeding					
Hammond et al. [43]	10 active men	Acute exercise	High-intensity running (8 × 5 min at 85% VO_2peak_) followed by steady-state running (60 min at 70% VO_2peak_) 3.5 h later. Steady-state running was either commenced with high or low (but high fat) CHO availability. Muscle glycogen was similar in both groups (200 mmol/kg dw) post-steady-state running	p70S6K activity was suppressed with high-fat feeding Similar gene expression of PGC-1α, p53, CS, Tfam, PPAR and ERRα in both groups	NA
Periodized model					
Impey et al. [48]	11 amateur male cyclists	Acute exercise	Based on the principle of ‘fuel for the work required’. 4 × 30 s HIT cycling (150% PPO) and 45 min steady-state cycling (50% PPO) followed by 1 min efforts (80% PPO) until exhaustion with either HIGH or LOW glycogen (by previous exercise/diet manipulation for 36 h previously). The HIGH group consumed CHO before, during and after exercise, whereas the LOW group consumed leucine-enriched protein	36 h of prior CHO restriction enhanced p53, SIRT1 and Tfam gene expression. CHO restriction before and during exercise induced work-efficient AMPK signalling. Post-exercise CHO restriction and keeping glycogen < 100 mmol/kg dw reduced p70S6K activity	Exercise capacity (1-min efforts at 80% PPO) enhanced in HIGH trial (158 vs. 100 min)
Burke et al. [45]	22 international male race walkers	3 weeks 7 × week	3 weeks of intensified training (race walking, resistance training, cross training). Athletes consumed three different diets across the training period: (a) high CHO; (b) LCHF; (c) periodized CHO intake with periods of low CHO training. Acute glycogen status not measured	NA	Similar improvements in VO_2peak_ between all groups Improved 10-km race times in the high CHO and periodized CHO groups (no change in LCHF) LCHF diet increased the O_2_ cost of race walking
Gejl et al. [55]	26 elite male endurance athletes	4 weeks 7 × week	4 weeks of intensified training. Athletes either performed all sessions with high CHO availability or followed a periodized model, performing three sessions per week with reduced CHO availability. Glycogen content was 400 mmol/kg dw following LOW carbohydrate availability training session	Similar increase in maximal CS activity No increase in β-HAD activity in either group	Similar improvement in VO_2max_ and 30-min TT performance between groups

Where possible, muscle glycogen status of the relevant experimental trials is also cited

*β*-*HAD* 3-hydroxyacyl-CoA dehydrogenase, *ACC* acetyl-CoA carboxylase, *AMPK* AMP-activated protein kinase, *BM* Body Mass, *CHO* carbohydrate, *CD36* cluster of differentiation 36, *CPT1* carnitine palmitoyltransferase 1, *CS* citrate synthase, *COX* cytochrome c oxidase, *ERRα* estrogen-related receptor α, *FABP3* fatty acid binding protein, *GLU* glucose, *GLUT4* glucose tr ansporter type 4, *HIT* high-intensity training, *HKII* hexokinase II, *IL* interleukin, *LCHF* low-carbohydrate, high-fat, *LIT* low intensity training, *LPL* lipoprotein lipase, *MAP* maximal aerobic power, *NA* not available, *NORM* normal, *NRF*-*1* nuclear respiratory factor 1, *p38MAPK* p38 mitogen-activated protein kinase, *p53* tumor protein 53, *p70S6K* ribosomal protein S6 kinase, *PDK4* pyruvate dehydrogenase kinase 4, *PGC*-*1α* peroxisome proliferator-activated receptor gamma coactivator 1-α, *PLA* placebo, *PM* post meridian, *PPAR* peroxisome proliferator-activated receptor, *PPO* peak power output, *SIRT1* NAD-dependent deacetylase sirtuin-1, *SDH* succinate dehydrogenase, *Tfam* transcription factor A, *TT* time trial, *TTE* time to exhaustion, *UCP3* uncoupling protein 3, *VO*_*2max*_ maximum rate of oxygen consumption, *VO*_*2peak*_ peak rate of oxygen consumption, *W*_*max*_ Watt maximum, *YoYoIR2* Yo-Yo intermittent recovery test 2

**Table 2:** Summary of key outcomes from train-low studies as categorized under the measures of cell signalling, gene expression, enzymatic changes and performance outcomes. Studies are presented according to those demonstrating positive changes, no change or negative changes

	Positive	No/equivalent change	Negative
Muscle (*n* = 25)			
Signalling (*n* = 11)	73% (*n* = 8) Steinberg et al. [21] Cochran et al. [22] Yeo et al. [23] Akerstrom et al. [27] Wojtaszewski et al. [36] Chan et al. [37] Bartlett et al. [38] Lane et al. [39]	27% (*n* = 3) Hammond et al. [43] Impey et al. [48] Lee-Young et al. [49]	0%
Gene expression (*n* = 12)	75% (*n* = 9) Pilegaard et al. [15a, b] Pilegaard et al. [16] Steinberg et al. [21] Psilander et al. [24] Chan et al. [37] Bartlett et al. [38] Lane et al. [39] Impey et al. [48]	25% (*n* = 3) Cochran et al. [22] Hammond et al. [43] Jensen et al. [54]	0%
Enzyme activity/protein content (*n* = 9)	78% (*n* = 7) Hansen et al. [9] Yeo et al. [17] Morton et al. [18] Hulston et al. [19] Van Proeyen et al. [30] De Bock et al. [31] Nybo et al. [32]	22% (*n* = 2) Cochran et al. [20] Gejl et al. [52]	0%
Physiological responses			
Lipid oxidation (*n* = 17)	47% (*n* = 8) Yeo et al. [17] Hulston et al. [19] Akerstrom et al. [27] Wojtaszewski et al. [36] Bartlett et al. [38] Lane et al. [39] Hammond et al. [43] Impey et al. [48]	53% (*n* = 9) Pilegaard et al. [15a] Marquet et al. [40, 41] Van Proeyen et al. [30] De Bock et al. [31] Nybo et al. [32] Burke et al. [45] Lee-Young et al. [49] Gejl et al. [52]	0%
Efficiency/economy (*n* = 2)	50% (*n* = 1) Marquet et al. [40]	50% (*n* = 1) Burke et al. [45]	
Performance			
Exercise performance changes (*n* = 11)	37% (*n* = 4) Hansen et al. [9] Cochran et al. [20] Marquet et al. [40, 41]	63% (*n* = 7) Yeo et al. [17] Morton et al. [18] Hulston et al. [19] Van Proeyen et al. [30] Nybo et al. [32] Burke et al. [45] Gejl et al. [52]	0%
Impaired training intensity (*n* = 3)	0%	0%	100% (*n* = 3) Yeo et al. [17] Hulston et al. [19] Lane et al. [56]

### Twice Per Day Training

On the basis that reduced pre- [15] and post-exercise [16] muscle glycogen availability augments expression of genes regulating substrate utilization and mitochondrial biogenesis, initial training studies adopted a ‘training twice every second day versus once daily’ research design. In this approach, subjects complete a morning training session to reduce muscle glycogen followed by several hours of reduced CHO intake so that the second training session of the day is commenced with reduced muscle glycogen. Using this model, 3–10 weeks of ‘train low’ increases oxidative enzyme activity [9, 17–19], whole body [17, 19] and intramuscular lipid utilization [19] and improves exercise capacity [9] and performance [20]. It is difficult to ascertain if the enhanced training response is mediated by the upregulation of transcriptional responses induced by CHO restriction in recovery from the morning exercise session and/or the enhanced cell signalling responses [21–24] associated with commencing the afternoon training session with reduced muscle glycogen. In addition to low glycogen availability, there may also be compounding effects of stacking training sessions in close temporal proximity where altering the mechanical, metabolic and hormonal environment may also modulate the enhanced training response. Notwithstanding a potential reduction in absolute training intensity in the afternoon session [17], the twice per day model provides a practical framework whereby the accumulative total time with reduced muscle glycogen is increased. Depending on the length of the interval between the first and second session (i.e. recover low) and the actual duration of the second training session (i.e. train low), the accumulated low glycogen period could range from 3 to 8 h.

### Fasted Training

Exercising fasted represents a simpler model of ‘train low’ whereby breakfast is consumed after a morning training session. In this model, pre-exercise muscle glycogen is not different between fasted or fed conditions, but liver glycogen and circulating glucose is higher during fed conditions. In contrast, increased free fatty acid (FFA) availability and lipid oxidation occur in fasted conditions when exercise is matched for intensity, duration and work performed [25]. Depending on the timing of CHO feeding in relation to the commencement of exercise (e.g. > 60 min before exercise versus < 10 min before and/or during exercise), such differences in FFA availability may manifest from the beginning of exercise [25] or not until after 30–40 min of exercise, respectively [26]. Exercising fasted increases AMP-activated protein kinase (AMPK) activity [27] and post-exercise gene expression [28, 29], while several weeks of fasted training increases oxidative enzyme activity [30], lipid transport protein content [31] and resting glycogen storage [32]. However, it is noteworthy that such adaptations are likely regulated via CHO restriction as opposed to true fasted conditions. Indeed, consuming approximately 20 g of whey protein before and during CHO-restricted training sessions still permits mobilization of FFAs [33] and activation of the AMPK signalling axis [34], while also improving net muscle protein balance [35].

### Sleep Low, Train Low

In the ‘sleep low, train low’ model, participants perform an evening training session, restrict CHO during overnight recovery, and then complete a fasted training session the following morning. The accumulative total time with reduced muscle glycogen could therefore extend to 12–14 h depending on the timing and duration of the training sessions and sleep period. Acute models of ‘sleep low, train low’ (where morning exercise is commenced with glycogen < 200 mmol/kg dw) enhance the activation of AMPK, p38 mitogen-activated protein kinase (p38) and tumour protein p53 (p53) signalling [36–38], although responses are attenuated if trained cyclists complete a morning steady-state session (thus suggesting that both intensity and training status are important) where pre- and post-exercise glycogen content is 350 and 250 mmol/kg dw, respectively [39]. Using a sleep-low model similar to that used by Lane and colleagues [39], Marquet et al. [40, 41] observed that 1–3 weeks of sleep-low training in elite triathletes and cyclists improves cycling efficiency (3.1%), 20 km cycling time-trial performance (3.2%) and 10 km running performance (2.9%) compared with traditional train-high approaches. At present, the extent of CHO restriction required to elicit conditions considered best representative of ‘sleep low’ are not well-defined. However, it is noteworthy that studies demonstrating beneficial effects on cell signalling [38] and performance adaptations [40, 41] have completely restricted CHO after the evening training session and after subjects were fed a protein-only meal or beverage. Nonetheless, similar to other train-low models, the sleep-low paradigm is subject to the obvious limitations of lack of a placebo controlled, double-blinded design. Such limitations are particularly relevant where performance improvements have been observed with only three sessions of ‘sleep low, train low’ [41].

### High-Fat Feeding

In addition to manipulating CHO availability, it is possible that associated elevations in circulating FFA availability also regulate relevant cell signalling pathways. Indeed, the exercise-induced activation of p38MAPK is suppressed with pharmacological ablation of FFA availability [42]. Nonetheless, while we acknowledge the potential role of acute FFA-mediated signalling (as occurring secondary to the primary intervention of CHO manipulation), it is unlikely that CHO restriction in combination with chronic high-fat feeding is beneficial for training adaptations. Indeed, 1–5 days of high-fat feeding reduces the expression [43] and activity of the pyruvate dehydrogenase (PDH) complex [44], ultimately impairing CHO oxidation and high-intensity performance. Furthermore, Burke et al. [45] observed that exercise economy and performance were negatively impacted in elite race walkers following 3 weeks of a high-fat diet when compared with periodized and high CHO availability. Emerging data also suggest that high-fat feeding may impair muscle protein synthesis [46], potentially via reduced activation of mammalian target of rapamycin (mTOR) and ribosomal protein S6 kinase (p70S6K) signalling [43].

### Amalgamation of Train-Low Paradigms and CHO Restriction-Induced Calorie Restriction

In the real-world environments of elite endurance athletes, it is likely that athletes practice an amalgamation of the aforementioned train-low paradigms (either through default of their current training structure or via coach and sport scientist-led practices), as opposed to undertaking one strategy in isolation. Additionally, elite athletes may also undertake 20–30 h of training per week, whereas many of the study designs reviewed thus far have utilized training programmes of < 10 h per week. The complexity of practical train-low models is exacerbated by observations that endurance athletes practice day-to-day or longer-term periods of energy periodization (as opposed to CHO per se) in an attempt to reduce body and fat mass in preparation for competition [10, 47] (Morton, unpublished observations). Indeed, the performance improvements observed by Marquet et al. [40] were also associated with a 1 kg reduction in fat mass induced by the sleep-low model. Notwithstanding reductions in body mass, it is indeed possible that many of the skeletal muscle adaptations associated with ‘train low’ are mediated by repeated and transient periods of energy restriction as opposed to CHO restriction per se. We observed that the post-exercise expression of peroxisome proliferator-activated receptor gamma coactivator 1-α (PGC-1α), p53, mitochondrial transcription factor A (Tfam) and peroxisome proliferator-activated receptor (PPAR) messenger RNA (mRNA) were elevated with similar magnitude and time-course when a low CHO and high-fat diet was consumed versus an isoenergetic high-CHO feeding strategy [43]. Such data conflict with previous observations from our laboratory [38], where post-exercise CHO and calorie restriction augments the expression of many of the aforementioned genes. Given the similarities in metabolic adaptation to both CHO and calorie restriction, such data raise the question as to whether the enhanced mitochondrial responses observed when training low are actually due to transient periods of calorie restriction (as mediated by a reduction in CHO intake) as opposed to CHO restriction per se. This point is especially relevant given that many endurance athletes present daily with transient periods of both CHO and calorie restriction due to multiple training sessions per day, as well as longer-term periods of suboptimal energy availability [47].

## The Glycogen Threshold Hypothesis: Muscle and Performance Adaptations Associated with CHO Restriction Occur Within a Range of Absolute Muscle Glycogen Concentrations

**Fig. 2:**
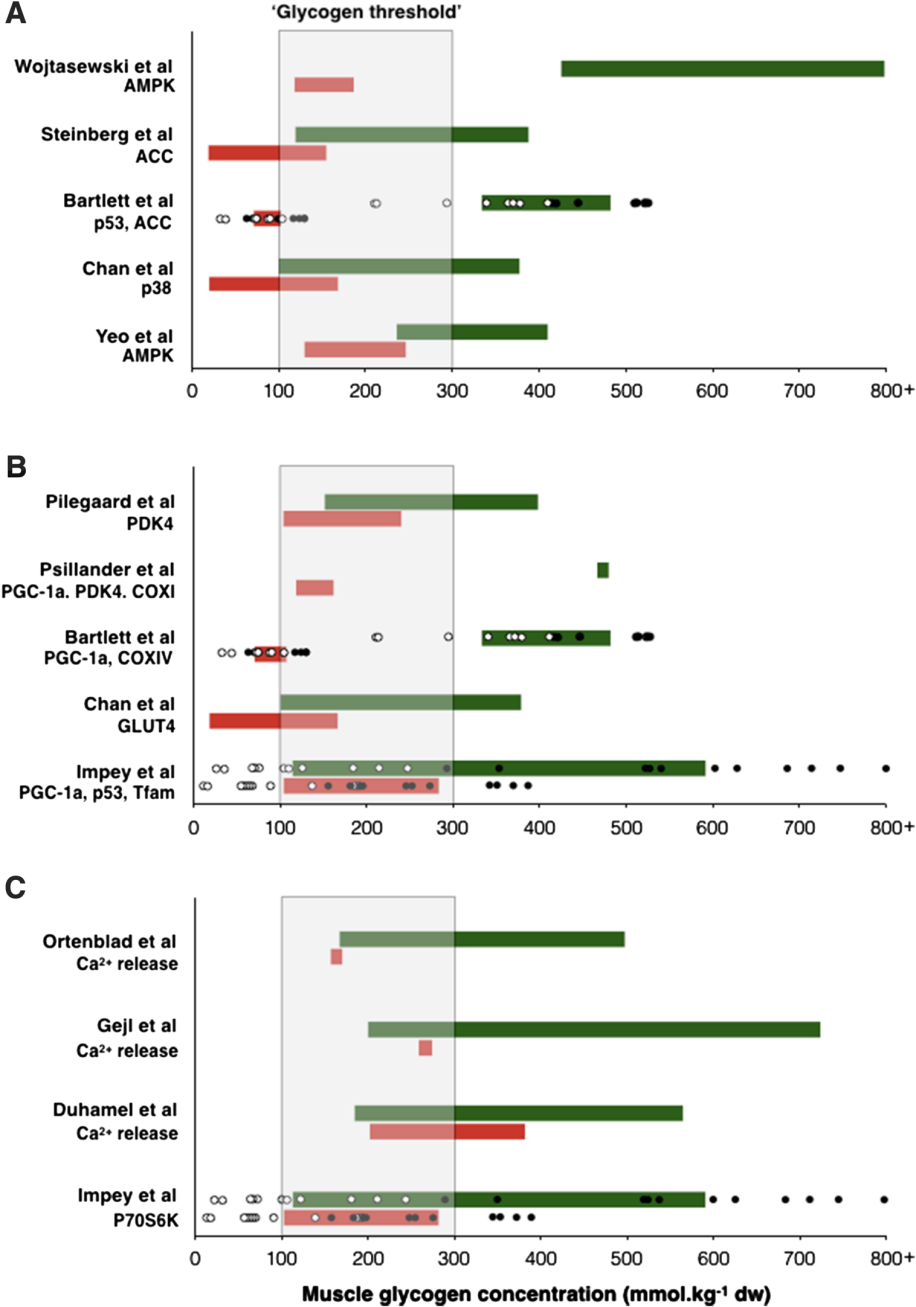
Overview of studies supporting the glycogen threshold hypothesis. Studies are categorized into those examining **a** cell signalling, **b** gene expression and **c** muscle contractile capacity and post-exercise signalling. In **a** and **b**, the green bars represent the trial within the specific study that has been completed with high muscle glycogen, and the red bars represent the trial completed with low muscle glycogen. The length of the bar in both instances corresponds to the pre- and post-exercise muscle glycogen concentration. Additionally, in studies from the authors’ laboratory (Bartlett et al. [38] and Impey et al. [48]), black and white circles represent individual subjects’ pre- and post-exercise muscle glycogen concentrations, respectively. In **c**, a variety of CHO manipulation protocols have been adopted to examine the effect of high (green bars) and low (red bars) muscle glycogen concentration on contractile properties and post-exercise cell signalling. The shaded area represents a potential muscle glycogen threshold in which exercise should be commenced (albeit specific to the training status of the participants studied in these investigations). *AMPK* AMP-activated protein kinase, *ACC* acetyl-CoA carboxylase, *Ca*^*2*+^ calcium, *COX* cytochrome c oxidase, *p38MAPK* p38 mitogen-activated protein kinase, *p70S6K* ribosomal protein S6 kinase, *PDK4* pyruvate dehydrogenase kinase 4, *PGC*-*1α* peroxisome proliferator-activated receptor gamma coactivator 1-α, *Tfam* mitochondrial transcription factor A

Given that the enhanced training response associated with ‘train low’ is potentially mediated by muscle glycogen availability, it is prudent to consider the absolute glycogen concentrations required to facilitate the response. In this regard, examination of available train-low studies (see Table 1) demonstrates that adaptations associated with CHO restriction are particularly evident when absolute pre-exercise muscle glycogen concentrations are ≤ 300 mmol/kg dw. However, restoring post-exercise glycogen levels to > 500 mmol/kg dw attenuates exercise-induced changes in gene expression [16], and keeping glycogen (and energy) at critically low post-exercise levels (i.e. < 100 mmol/kg dw) may reduce the regulation of protein synthesis pathways [48]. CHO feeding during exercise also attenuates AMPK-mediated signalling but only when glycogen sparing occurs [27, 49]. However, it should be noted that commencing exercise with < 200 mmol/kg dw is likely to impair training intensity due to the lack of muscle substrate and impact on the contractile capacity of muscle cells via impaired calcium regulation [50–52]. Additionally, repetitive daily training in the face of a reduction in CHO availability (so as to reduce pre-exercise muscle glycogen concentration) may increase susceptibility to illness [53]. On this basis, such data suggest the presence of a muscle glycogen threshold whereby a critical absolute level of glycogen depletion must be induced for significant activation of cell signalling pathways to occur on the proviso, of course, that the desired training workload and intensity can be completed without any maladaptations. In this way, the glycogen threshold provides a metabolic window of muscle glycogen concentrations (e.g. 300–100 mmol/kg dw) that is permissive to facilitating the required training intensity, acute cell signalling responses and post-exercise remodelling processes (see Fig. 2). Nonetheless, the potential presence of a glycogen threshold is not to say that individuals will not adapt to endurance training if they do not exercise within a suggested threshold. Rather, such a threshold illustrates the possibility that the enhanced adaptations associated with ‘train low’ are especially prominent once a certain level of glycogen depletion has occurred. We also acknowledge the between-subject variations in glycogen depletion that is often observed within the literature, and how this may also influence any potential glycogen threshold range (individual subject responses are also displayed in Fig. 2 for the data presented from the authors’ laboratory).

## Fuel for the Work Required: Practical Application of the Glycogen Threshold Hypothesis

The studies reviewed thus far have typically adopted matched work exercise protocols whereby CHO availability is manipulated prior to training sessions consisting of identical intensity, duration and work performed. More recently, we also adopted an experimental design in which participants completed an exhaustive exercise capacity test in conditions of high or low pre-exercise muscle glycogen (600 and 300 mmol/kg dw, respectively) [48]. All participants cycled for longer in the high trial versus the low trial, with average exercise capacity increased by 60 min. We observed identical cell signalling and post-exercise gene responses between trials (e.g. AMPK-PGC-1α) despite the completion of less work performed in the low glycogen trial. However, it is acknowledged that to stimulate other endurance-related adaptations (e.g. central changes such as cardiac hypertrophy), high glycogen conditions would be superior, given that it would allow for the completion of more work performed and, of course, an elevated heart rate for a much longer training duration. Moreover, at the muscular level it appears that commencing exercise with high muscle glycogen requires the completion of considerably more work in order to elicit comparable cell signalling and muscle remodelling responses than those occurring when exercise is commenced with low muscle glycogen. In this situation, it is suggested that subjects eventually surpass a critical level of glycogen depletion, thereby completing a proportion of exercise within the glycogen threshold required to stimulate cell signalling. Given that elite endurance athletes rarely complete identical sessions or train with maximal intensity (or to exhaustion) on consecutive training sessions, either within or between days, such data support the concept of ‘fuelling for the work required’, whereby CHO availability is adjusted in accordance with the demands of the specific training session to be completed. In this way, absolute daily CHO intake and distribution can be modified so that key sessions are performed within or close to the suggested muscle glycogen threshold. As initially alluded to by Sherman et al. [6], the question that emerges therefore is not how much an athlete can super-compensate their glycogen stores but rather does the athlete’s diet contain enough CHO to maintain training intensity while also creating a consistent metabolic milieu that is conducive to facilitating training adaptations. Using the sport of road cycling, we present a theoretical overview of the model in Table 3.

**Table 3:**
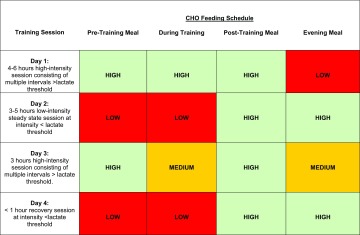
Theoretical overview of the ‘fuel for the work required’ model

The model is presented for an elite endurance athlete (e.g. road cyclist) who trains once per day on 4 consecutive days where each session commences at 10:00 am each day. In this example, the athlete has four main feeding points and the CHO content of each timepoint is colour coded according to a red, amber or green (RAG) rating that represents low, medium and high CHO intake. Note that we have not prescribed specific CHO quantities and deliberately chose a RAG rating so as to highlight the necessity for flexibility in relation to athlete history, training status and specific training goals, etc. Rather, the model is simply presented to illustrate how train-low paradigms can be amalgamated to adjust CHO availability day-by-day and meal-by-meal according to the demands of the specific training session across the 4-day training block. In this example, high CHO intake is advised before, during and after the training session on day 1 (e.g. ‘train high’), but reduced in the evening meal to facilitate sleep low and train low for a lower-intensity session on day 2 (i.e. likely commenced with reduced muscle glycogen and withholding or reducing CHO content of the pre-training meal). Following completion of the second training session, high CHO availability is prescribed for the remainder of day 2 so as to promote glycogen storage in preparation for a higher absolute workload and intensity on day 3. Given that day 4 is a designated recovery day of much lower duration and intensity, CHO intake is then reduced in the evening of day 3 and breakfast of day 4, but is then increased throughout the remainder of day 4 in order to prepare for another 4-day training block. The model should be adjusted according to the number of feeding points and training sessions that are to be undertaken on each day, e.g. a marathon runner [7] would have a different CHO periodization model than a cyclist. Careful day-to-day periodization in a meal-by-meal manner (as opposed to chronic periods of CHO restriction or CHO feeding) is likely to maintain metabolic flexibility and still allow the completion of high-intensity and prolonged duration workloads on heavy training days, e.g. interval-type sessions undertaken above lactate threshold. Intuitively, ‘train low’ may be best left to those training sessions that are not CHO-dependent and where the intensity and duration is not likely to be compromised by reduced CHO availability (e.g. steady-state-type training sessions performed at intensities below the lactate threshold). Additionally, the model may also provide a framework to aid body mass loss given that train-low sessions on lower-intensity training days may allow for the creation of energy deficits without negating training intensity

*CHO* carbohydrate

## Critical Reflections and Limitations on Practical Application of the Glycogen Threshold Hypothesis

**Fig. 3:**
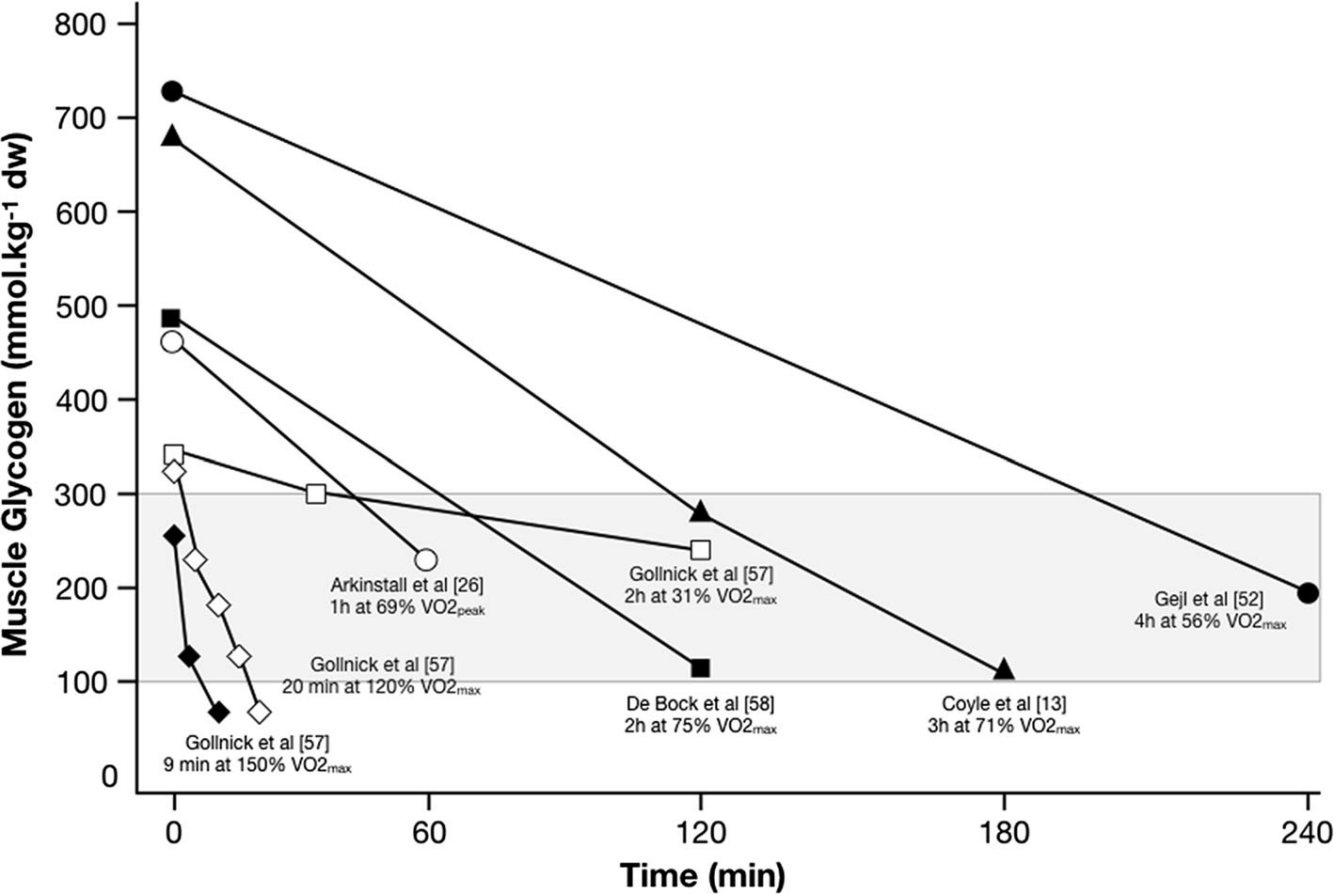
Muscle glycogen utilization according to studies incorporating varied exercise intensity, duration, and pre-exercise muscle glycogen concentration. Such data illustrate how the pattern of glycogen use can vary (according to the interactive effects of the aforementioned parameters) and how this should be considered in relation to the proposed glycogen threshold (shaded area). Data represent a sampling from studies compiled from cycling exercise protocols only and represent glycogen use in the vastus lateralis muscle

Despite the theoretical rationale for periodic train-low sessions and the potential presence of a glycogen threshold, there are many obvious challenges in bringing this to life in applied practice. Crucially, our understanding of the glycogen and CHO cost of the habitual training sessions undertaken by elite athletes is not well known, thus limiting our ability to prescribe training sessions that may elicit any glycogen threshold. Indeed, rates of glycogen utilization are not linear and are dependent on substrate availability, training status, intensity and duration, all of which can modify the regulation of glycogenolysis via hormonal control or allosteric regulation. Furthermore, any potential glycogen threshold is also likely to be training-status-specific and heavily dependent on the characteristics of the exercise modality in question, e.g. a lower absolute threshold may be present for a metabolically based sport such as cycling (non-weight-bearing activity with a lower eccentric load), whereas a higher threshold may be present in running, given that weight bearing, eccentric contractions and higher neuromuscular loads are present. While examination of relevant studies (see Fig. 3 for a sampling of experimental data from cycling studies) provides some indication of the intensity and duration (against a background of differences in pre-exercise glycogen status) that would be required to sufficiently deplete an athlete’s glycogen close to or within the glycogen threshold, considerable assumptions must be made. Indeed, given the lack of technology available to non-invasively quantify muscle glycogen, practical application of the glycogen threshold hypothesis relies heavily on practitioners’ theoretical knowledge of glycogen utilization with their specific sport and, moreover, detailed assessment of an athlete’s previous day(s) training loads and macronutrient intake in order to formulate appropriate guidance for future training sessions. To this end, awareness of relevant sport-specific literature coupled with both external (e.g. power outputs, running velocities) and internal (e.g. heart rate, blood lactate, ratings of perceived exertion) assessment of training load can be collectively used in conjunction with laboratory assessments of substrate utilization so as to estimate the potential CHO cost of real-world training sessions. Considerable debate is also likely to exist among researchers and practitioners as to what amount of dietary CHO actually equates to the red, amber and green (RAG) ratings outlined in Table 3, and, undoubtedly, further sport-specific studies are warranted.

## Conclusions

The emergence of CHO availability (specifically muscle glycogen concentration) as a regulator of training adaptation is now an accepted area of research that has practical implications for athlete training strategies. While this is a hot topic among athletes, coaches and sport scientists, the optimal periodization strategy to implement periods of CHO restriction into an overall training programme is not well understood. Furthermore, practical models of CHO (and energy) periodization are likely to be highly specific to the training structure and culture of the sport in question, as well as the athlete’s specific training goals. Accordingly, we have merely presented a hypothetical framework of fuelling for the work required to illustrate how isolated train-low models can be amalgamated to produce day-by-day and meal-by-meal manipulation of CHO availability. While we readily acknowledge the requirement for sport-specific models and long-term training studies (especially to demonstrate if train-low muscle adaptations actually correspond to meaningful changes in exercise performance), we also pose several fundamental questions that are relevant across many sporting models. First, does the presence of a ‘graded’ muscle glycogen threshold really exist, and, if so, how is this affected by training status? Second, should train-low sessions always be left to low-intensity-type sessions or is it the deliberate completion of a high-intensity session (even at the expense of a potential reduction in absolute workload) that is really required to create the metabolic milieu that is conducive to signalling? Third, what is the minimal CHO intake and glycogen concentration required to facilitate periods of ‘train low’ without compromising absolute training intensity during specific sessions? Fourth, is the enhanced training response associated with ‘train low’ regulated by CHO and/or energy restriction, and, if the latter, how do we periodize and structure such training interventions without inducing maladaptations? Finally, are there novel molecular targets and protein modifications/localization that also contribute to the regulation of nutrient and exercise sensitive pathways? When considered this way, it is remarkable that the study of only 500 g of substrate (the approximate whole-body storage of CHO) remains as exciting as ever.
